# Cardiac and testicular toxicity effects of the latex and ethanolic leaf extract of *Calotropis procera* on male albino rats in comparison to abamectin

**DOI:** 10.1186/s40064-016-3326-7

**Published:** 2016-09-22

**Authors:** Osama M. Ahmed, Hanaa I. Fahim, Magdy W. Boules, Heba Y. Ahmed

**Affiliations:** 1 Physiology Division, Zoology Department, Faculty of Science, Beni-Suef University, Beni Suef, Egypt; 2 Rodents Division, Harmful Animals Department, Plant Protection Research Institute, Agriculture Research Center, Giza, Egypt

**Keywords:** *Calotropis procera*, Abamectin, Heart, Testis, Toxicity

## Abstract

The present study aims to assess the toxic effect of latex and ethanolic leaf extract of *Calotropis procera* (*C. procera*), in comparison to abamectin, on serum biomarkers of function and histological integrity of heart and testis in male albino rats. To achieve this aim, the albino rats were separately administered 1/20 and 1/10 of LD_50_ of *C. procera* latex, ethanolic *C. procera* leaf extract and abamectin respectively by oral gavage for 4 and 8 weeks. *C. procera* latex and leaf extract as well as abamectin markedly elevated the activities of serum CK-MB, AST and LDH at the two tested periods in a dose dependent manner. Lipid peroxidation was significantly increased while GSH level and GPx, GST and SOD activities were significantly depleted in heart and testis of all treated rats. All treatments also induced a marked increase in serum TNF-α and decrease in serum IL-4, testosterone, FSH and LH levels in a dose dependent manner. The latex seemed to be more effective in deteriorating the testicular function and sex hormones’ levels while the ethanolic leaf extract produced more deleterious effects on oxidative stress and antioxidant defense system in both heart and testis. The normal histological architecture and integrity of the heart and testis were perturbed after treatments and the severity of lesions, which include odema, inflammatory cell infiltration, necrosis and degeneration, is dose and time dependent. In conclusion, the findings of this study indicated that *C. procera* latex and ethanolic extract of leaves could induce marked toxicity in heart and testis and these toxic effects may be more or less similar to those of abamectin. The cardiotoxicity and testicular toxicity may be mediated via stimulation of inflammation, increased oxidative stress and suppression of antioxidant defense system.

## Background

Development of new rodent control methodologies and strategies continues to be an exciting subject for researchers. In the last two decades, there has been a shift in rodenticide use, with researchers and pest control practitioners taking a renewed interest in alternatives to anticoagulants; this stems from increased resistance of pests to 1st generation anticoagulants (Quy et al. 1995), as well as concerns over the secondary-poisoning risks and wildlife contamination associated with field use of 2nd generation anticoagulants (Eason and Turck 2002; Thomas et al. 2011). It was found by Carvalho et al. (2003) that many natural compounds have been suggested as alternatives to conventional chemicals used for pest control. It was reported that plant extracts have been used as pesticides by humans (Abou-Hashem 2013). The use of toxic plants is especially prevalent in the developing and underdeveloped countries, where plants grown locally are cheaper than the synthetic chemical pesticides (EL-Gengaihi et al. 1997; Paul and Kumar 2009; Million et al. 2010).

*Calotropis* is a small genus having six species of shrubs or small trees, distributed in tropical and subtropical Africa, Asia and America. Two species namely *Calotropis procera* (*C. procera*) and *Calotropis gigantea* (*C. gigantea*) are found in India which closely resembled to each other in structure and in functional uses (Bhatnagar 1950). It was revealed that *C. procera* includes various chemicals which are useful for various activities (Sheth 2011; Begum et al. 2013). The entire plant has been reported to contain alkaloids, sterols, flavonoids, cardiac glycosides, triterpenoids and usharin (Suresh Kumar et al. 2013). In an earlier study, various medicinal properties such as a laxative, anthelmintic, purgative, anti-inflammatory and diuretic have been documented (Iqbal et al. 2005). Different parts of *C. procera* and its latex have shown analgesic, antibacterial and wound healing properties in traditional medicine (Laitiff et al. 2010; Lima-Filho et al. 2010). The previous pharmacological studies on *C. procera* include reports of its anticancer, antifungal and insecticidal activity (Ahmed et al. 2006; Hassan et al. 2006).

Despite these uses, *C. procera* poses varying toxic effects in animals through air borne allergies, touch and consumption in livestock. Vadlapudi and Naidu (2010) revealed that the plant is also known for its toxic properties that include iridocyclites, dermatitis and acts like a poison and produces lethal effects. Toxicity of *C. procera* is reported in sheep in the form of anorexia and diarrhea. Consumption of this plant leads to severe poisoning to livestock as well as man. Incidental ingestion of fresh *C. procera* leaves has been suggested as toxic to many ruminants by several farmers from the Brazilian semi-arid region. These observations are supported by some studies that have reported toxic effects promoted by *C. procera* latex and leaves (Mahmoud et al. 1979a, b; Singhal and Kumar 2009). The latex of *C. procera* contains several cardenolides such as calotropin, catotoxin, calcilin and gigantin which are caustic and considered poisonous in nature (Kuriachen and Dave 1989).

Biocides are widely used in agriculture and can contaminate rivers and other water bodies due to transport from cultivated areas (Cerejeira et al. 2003; Maloschik et al. 2007). Abamectin, the non-proprietary name assigned to avermectin B1, is a mixture of two components, with the major component avermectin B1a 80 % of the mixture, and the minor component avermectin B1b, 20 % of the mixture, differing by a single methylene group (Agarwal 1998). The two components, B1a and B1b, have similar biological and toxicological properties (Lankas and Gordon 1989; Gallo and Lawryk 1991). As indicated by Kolar et al. (2008), abamectin has been used in several countries as a pest control agent in livestock and as an active substance of nematicides and insecticides for agricultural use. ABM may be valuable in agriculture; it may be highly toxic to mammals (Moline et al. 2000).

Therefore, this study aims to verify the toxic effect of latex and ethanolic extract of leaves of *C. procera* on heart and testis compared with the biocide abamectin.

## Methods

### Plant materials

The leaves and latex of *C. procera* were obtained from East desert of Beni-Suef Governorate. The plant was authenticated by Dr. Walaa Azmy Hasan, lecturer of plant taxonomy, Department of Botany, Faculty of Science, Beni Suef University.

### Collection of leaves and extract preparation

Only mature leaves without sign of lesion were used. The leaves of *C. procera* were extracted by ethanol according to Freedman et al. (1979). Leaves were washed with distilled water, air dried at room temperature and ground into fine powder using electrical mixer. Five hindered grams of the powder were suspended in 1 l of ethanol 95 % for 72 h then filtered and the filtrate was evaporated by rotary evaporator at high pressure and temperature 40–50 °C at Faculty of pharmacy, Beni-Suef University, Egypt. The extract was kept in a refrigerator at −30 °C until use.

### Latex collection

Fresh latex was obtained by breaking the leaf stock and allowing the latex to flow into a glass beaker. It was freshly prepared before injection.

### Pesticide and chemicals

Abamectin (1.8 % EC) is a mixture of 80 % avermectin B1a and maximum of avermectin B1b used as an acaricide. It was obtained from Synganta Agro. Co. (Switzerland).

Reagent kits used for determination of creatinine kinase-MB (CK-MB) activity was purchased from Spinreact Company (Spain). Aspartate aminotransferase (AST) and lactate dehydrogenase (LDH) reagent kits were purchased from Biosystems Company (Spain). Testosterone reagent kits were purchased from BioSource Company (Belgium). Follicle stimulating hormone (FSH) and luteinizing hormone (LH) reagent kits were purchased from Monobind, INC. (USA). Tumor necrosis factor-alpha (TNF-α) and Interleukin-4 (IL-4) kits were purchased from R&D Systems, Inc. (USA). All other used chemicals are of analytical grade and were obtained from Sigma-Aldrich Chemical Company (USA).

### Experimental animals

Male albino rats weighing 120–150 g (8–10 weeks of age) were used as experimental animals in this investigation. They were obtained from the Animal House of Research Institute of Ophthalmology, Giza, Egypt. Animals were supplied daily standard pellet diet and were given water ad libitum. The animals were housed in polypropylene cages with good aerated stainless steel in Animal House of Zoology Department, Faculty of Science, Beni-Suef University, Egypt at temperature 20–25 °C and 12-h daily light dark cycles. The animals were kept for 2 weeks under observation before the onset of the experiment to exclude any intercurrent infection. All animal procedures are in accordance with the recommendation of the Experimental Animals Ethics Committee of Faculty of Science, Beni-Suef University. All efforts were done to decrease the suffering of animals to a minimum.

### Experimental design

Experimental animals were divided into seven groups as follow:

- Group 1: Rats of this group are regarded as control group and were administered 1 % carboxy methyl cellulose (CMC) by oral gavage for 4 and 8 weeks.
- Group 2: Rats of this group were orally administered 1/20 of LD_50_ (50 % lethal dose) of *C. procera* latex (66 µl/kg b. wt), dissolved in 1 % CMC, for 4 and 8 weeks. LD_50_ of *C. procera* latex is 1.316 ml/kg b. wt as detected by Fahim et al. (2016).
- Group 3: Rats of this group were orally administered 1/10 of LD_50_ of *C. procera* latex (132 µl/kg b. wt), dissolved in 1 % CMC, for 4 and 8 weeks.
- Group 4: Rats of this group were orally administered 1/20 of LD_50_ of ethanolic extract of *C. procera* leaves (4.78 mg/kg b. wt), dissolved in 1 % CMC, for 4 and 8 weeks. LD_50_ of ethanolic extract of *C. procera* leaves is 95.52 mg/kg b. wt (El-Shafey et al. 2011).
- Group 5: Rats of this group were orally administered 1/10 of LD_50_ of ethanolic extract of *C. procera* leaves (9.56 mg/kg b. wt), dissolved in 1 % CMC, for 4 and 8 weeks.
- Group 6: Rats of this group were orally administered 1/20 of LD_50_ of abamectin (vertimec 1.8 % EC) (0.44 mg/kg b. wt), dissolved in 1 % CMC, for 4 and 8 weeks. LD_50_ of abamectin vertimec (1.8 % EC) is 8.7 mg/kg body weight (Lankas and Gordon 1989; El-Shafey et al. 2011).
- Group 7: Rats of this group were orally administered 1/10 of LD_50_ of abamectin (vertimec 1.8 % EC) (0.87 mg/kg b. wt), dissolved in 1 % CMC, for 4 and 8 weeks.

### Samples preparation

At the end of the 4th and 10th weeks, six animals of each group were sacrificed under diethyl ether anesthesia. Blood samples were obtained from cervical vein, left to coagulate at room temperature and then they were centrifuged at 3000 rpm for 30 min. The clear non-haemolysed supernatant sera were quickly removed and were divided into 3 portions. The obtained samples were kept in deep freezer at −30 °C till used. Half of heart and testis were excised and removed quickly, homogenized by using in isotonic solution (0.9 % NaCl) and kept in deep freezer at −30 °C till used. The other half of heart and other testis were immediately excised and fixed in 10 % neutral buffered formalin for histopathological processing.

### Biochemical investigations

#### Detection of serum parameters related to heart function

Serum CK-MB activity was determined according to the method of Gerhart and Waldenström (1979). Serum AST and LDH activities were detected according to the methods of Gella et al. (1985) and Young (2000) respectively.

#### Assay of male sex hormones levels

Concentrations of FSH and LH in serum were detected according to the method of Odell et al. (1968) and Braunstein et al. (1976). Serum testosterone concentration was determined according to the method of Andreyko et al. (1986).

#### Assays TNF-α and IL-4 levels

Serum TNF-α and IL-4 levels were determined by the quantitative sandwich enzyme immunoassay technique according to the methods of Howard and Harada (1994) and Croft et al. (2012) respectively.

#### Assay of oxidative stress and antioxidant defense markers

Heart and testis glutathione content and lipid peroxidation were determined according to the methods of Beutler et al. (1963) and Preuss et al. (1998) respectively. Glutathione peroxidase (GPx), glutathione-*S*-transferase (GST) and superoxide dismutase (SOD) activities in heart and testis were assayed according to the methods of Matkovics et al. (1997), Mannervik and Gutenberg (1981) and Marklund and Marklund (1974) respectively.

### Statistical analysis

The data obtained from the experiment were analyzed using the one-way analysis of variance (ANOVA) (Roa et al. 1985) followed by LSD test to compare various groups with each other. Results were expressed as mean ± standard error (SE) and values of P > 0.05 were considered non-significantly different while those of P < 0.05 and P < 0.01 were considered significant and highly significant respectively. F-probability expresses the general effect between groups. Multi-factor analysis of variance (MANOVA) was also performed to evaluate the effect of time, dose and time–dose interaction.

## Results

### Effect on levels of serum parameters related to heart function

Data showing the effects of latex and ethanolic extract and ABM pesticide on serum markers of heart function are represented in Tables 1 and 2. The treatments of normal rats with latex and ethanolic extract as well as ABM for 4 weeks induced a highly significant increase (P < 0.01; LSD) in the activities of CK-MB and LDH. With the exception of the effect of 1/20 LD_50_ of ABM, serum AST and LDH activities were significantly increased (P < 0.01; LSD) after administration of the three tested materials for 8 weeks. One-way ANOVA (Table 1) depicted that the general effect between groups on serum CK-MB, AST and LDH activities was very highly significant (P < 0.001; F-probability). On the other hand, two-way ANOVA of normal–latex effect showed that the effect of dose was very highly significant (P < 0.001; F-probability) on CK-MB, AST and LDH activities while the time of administration had a significant effect (P < 0.05; F-probability) on CK-MB and insignificant effect (P > 0.05; F-probability) on AST and LDH activities. The dose–time interaction had insignificant effect (P > 0.05; F-probability) on CK-MB, AST and LDH activities. Regarding normal–extract effect, the dose, time and dose–time interaction had very highly significant effect (P < 0.001; F-probability) on CK-MB and LDH activities while the effect of dose and time on AST activity were very highly significant and significant respectively. Similar to latex and ethanolic extract effect, the dose effect of ABM was very highly significant (P < 0.001; F-probability). The time had insignificant effect (P > 0.05; F-probability) on CK-MB and AST activities and very highly significant effect (P < 0.001; F-probability) on LDH activity with ABM treatment. The effect of dose–time interaction was very highly significant on LDH activity due to ABM administration.

**Table 1:** Effect of latex and ethanolic leaf extract and abamectin on serum CK-MB, AST and LDH activities of normal rats

Treatments	Parameter					
	CK-MB (U/L)		AST (U/L)		LDH (U/L)	
	4 weeks	8 weeks	4 weeks	8 weeks	4 weeks	8 weeks
Vehicle (CMC 1 %) control	93.26 ± 5.49^f^	94.99 ± 4.70^f^	96.57 ± 2.55^fg^	86.65 ± 6.01^g^	749.58 ± 80.83^g^	738.89 ± 80.25^g^
1/20 LD_50_ latex	178.16 ± 21.79^bcde^	103.37 ± 18.49^f^	124.68 ± 5.48^abcd^	116.64 ± 2.27^bcde^	2897.51 ± 310.79^bc^	2775.36 ± 146.39^c^
1/10 LD_50_ latex	217.63 ± 25.89^b^	185.50 ± 18.35^bcde^	138.67 ± 5.95^a^	126.66 ± 10.24^ab^	3488.61 ± 182.80^a^	3471.67 ± 25.63^a^
1/20 LD_50_ ethanolic extract	177.62 ± 16.82^bcde^	135.00 ± 3.09^ef^	109.43 ± 1.59^cdef^	105.98 ± 3.26^ef^	3341.78 ± 296.19^ab^	1533.90 ± 23.68^f^
1/10 LD_50_ ethanolic extract	366.10 ± 31.39^a^	140.34 ± 3.69^def^	140.50 ± 6.32^a^	125.98 ± 6.36^ab^	3457.25 ± 325.48^a^	2085.50 ± 223.34^de^
1/20 LD_50_ ABM	194.03 ± 20.98^bcd^	142.40 ± 16.58^cdef^	108.87 ± 1.32^def^	99.45 ± 8.72^fg^	2195.30 ± 141.03^d^	1283 ± 10.57^fg^
1/10 LD_50_ ABM	195.09 ± 21.01^bc^	201.76 ± 23.23^b^	111.10 ± 4.70^bcdef^	125.34 ± 3.99^abc^	3194.32 ± 256.22^abc^	1581 ± 81.17^ef^
F-probability	P < 0.001		P < 0.001		P < 0.001	
LSD at 5 % level	53.828		16.0947		549.503	
LSD at 1 % level	72.491		21.675		735.985	

Data are expressed as mean ± SE

Number of animals in each group is six

For each parameter, means, which do not share the same superscript symbol(s), are significantly different at P < 0.05

**Table 2:** Analysis of variance for CK-MB, AST and LDH activities in serum of normal and treated rats

Source of variation	F-probability		
	CK-MB (U/L)	AST (U/L)	LDH (U/L)
A—Normal–latex effect			
Dose	P < 0.001	P < 0.001	P < 0.001
Time	P < 0.05	P > 0.05	P > 0.05
Dose–time	P > 0.05	P > 0.05	P > 0.05
B—Normal–extract effect			
Dose	P < 0.001	P < 0.001	P < 0.001
Time	P < 0.001	P < 0.05	P < 0.001
Dose–time	P < 0.001	P > 0.05	P < 0.001
C—Normal–Abamectin Effect			
Dose	P < 0.001	P < 0.001	P < 0.001
Time	P > 0.05	P > 0.05	P < 0.001
Dose–time	P > 0.05	P < 0.05	P < 0.001

### Effect on male sex hormones levels

Serum testosterone level was highly significantly reduced (P < 0.01; LSD) in male rats ingested latex, ethanolic extract and ABM for 4 and 8 weeks; the effect seemed to dose dependent. Treatments with 1/10 LD_50_ of the tested materials for 4 weeks induced a highly significant decrease (P < 0.01) in LH level while 1/20 extract only produced a significant effect at the same experimental period. With regard to FSH, the administration of the high dose of latex and extract significantly (P < 0.01; LSD) decreased serum FSH level after 4 weeks whereas the high dose of the three tested treatments induced a significant depletion (P < 0.01; LSD) of FSH level at the 8th week (Table 3). Concerning one way ANOVA, it was found that the general effect between groups on serum testosterone, FSH and LH concentrations was very highly significant (P < 0.001; F-probability) throughout the experiment (Table 3). Two-way ANOVA (Table 4) stated that the dose effect of latex was very highly significant (P < 0.001; LSD) on testosterone, FSH and LH while the time had very highly significant effect (P < 0.001; F-probability) on FSH level, highly significant effect (P < 0.01; F-probability) on LH level and insignificant effect (P > 0.05; F-probability) on testosterone level. The dose–time interaction had insignificant effect (P > 0.05; F-probability) on the three tested hormones. Concerning extract effect, the dose had very highly significant effect (P < 0.001; F-probability) on testosterone and FSH levels and highly significant effect (P < 0.01; F-probability) on LH level. Time had significant effect (P < 0.05; F-probability) on FSH level and insignificant effect significant effect (P > 0.05; F-probability) on testosterone and LH levels. The interaction between dose and time had insignificant effect (P > 0.05; F-probability) on the three tested hormones. Regarding ABM effect, dose had very highly significant effect (P < 0.001; F-probability) on testosterone and LH levels and highly significant effect (P < 0.01; F-probability) on FSH level. Time had significant effect (P < 0.05; F-probability) on testosterone and LH levels and very highly significant effect (P < 0.001; F-probability) on FSH level. The dose–time interaction had insignificant effect (P > 0.05; F-probability) on the three tested hormones.

**Table 3:** Effect of latex and ethanolic leaf extract and abamectin on serum testosterone, FSH and LH levels in normal rats

Treatments	Parameter					
	Testosterone (ng/ml)		FSH (mlU/ml)		LH (mlU/ml)	
	4 weeks	8 weeks	4 weeks	8 weeks	4 weeks	8 weeks
Vehicle (CMC1 %) control	2.71 ± 0.28^a^	2.22 ± 0.35^a^	0.26 ± 0.01^a^	0.23 ± 0.01^abc^	0.19 ± 0.01^a^	0.17 ± 0.01^abcd^
1/20 LD_50_ latex	1.21 ± 0.11^bcd^	1.1 ± 0.16^bcd^	0.25 ± 0.01^a^	0.20 ± 0.01^defg^	0.18 ± 0.01^ab^	0.14 ± 0.01^fg^
1/10 LD_50_ latex	0.75 ± 0.13^d^	0.84 ± 0.04^cd^	0.19 ± 0.01^efg^	0.18 ± 0.01^fg^	0.15 ± 0.01^efg^	0.14 ± 0.01^g^
1/20 LD_50_ ethanolic extract	1.63 ± 0.21^b^	1.38 ± 0.36^bc^	0.23 ± 0.01^abcd^	0.21 ± 0.01^cdef^	0.16 ± 0.01^cdef^	0.16 ± 0.01^bcde^
1/10 LD_50_ ethanolic extract	1.19 ± 0.06^bcd^	0.88 ± 0.16^cd^	0.20 ± 0.01^efg^	0.19 ± 0.02^efg^	0.16 ± 0.01^def^	0.14 ± 0.01^efg^
1/20 LD50 ABM	1.02 ± 0.15^cd^	0.81 ± 0.11^d^	0.25 ± 0.01^abc^	0.22 ± 0.01^bcde^	0.18 ± 0.01^ab^	0.18 ± 0.01^abc^
1/10 LD50 ABM	0.88 ± 0.07^cd^	0.68 ± 0.06^d^	0.24 ± 0.02^ab^	0.18 ± 0.02^g^	0.16 ± 0.01^cdef^	0.15 ± 0.01^efg^
F-probability	P < 0.001		P < 0.001		P < 0.001	
LSD at 5 % level	0.5479		0.0311		0.0199	
LSD at 1 % level	0.7379		0.0419		0.0269	

Data are expressed as mean ± SE

Number of animals in each group is six

For each parameter, means, which do not share the same superscript symbol(s), are significantly different at P < 0.05

**Table 4:** Analysis of variance for testosterone, FSH and LH concentrations in serum of normal and treated rats

Source of variation	F-probability		
	Testosterone (ng/ml)	FSH (mlU/ml)	LH (mlU/ml)
A—Normal–latex effect			
Dose	P < 0.001	P < 0.001	P < 0.001
Time	P > 0.05	P < 0.001	P < 0.01
Dose–time	P > 0.05	P > 0.05	P > 0.05
B—Normal–extract effect			
Dose	P < 0.001	P < 0.001	P < 0.01
Time	P > 0.05	P < 0.05	P > 0.05
Dose–time	P > 0.05	P > 0.05	P > 0.05
C—Normal–abamectin effect			
Dose	P < 0.001	P < 0.01	P < 0.001
Time	P < 0.05	P < 0.001	P < 0.05
Dose–time	P > 0.05	P > 0.05	P > 0.05

### Effect on serum TNF-α and IL-4 levels

Data represented in Tables 5 and 6 depicted that all treatments induced strong adverse effects on the normal levels of serum TNF-α and IL-4 of normal rats. Administrations of latex, ethanolic extract of *C. procera* and ABM for 4 and 8 weeks induced a highly significant increases (P < 0.01; LSD) in serum levels of TNF-α. On the other hand, while the higher doses of the ethanolic extract and ABM induced a highly significant effect on serum level of IL-4 after 4 weeks, the latex produced a significant effect as a result of the two tested doses after 4 and 8 weeks. ABM seemed to be the most effective in increasing serum TNF-α while latex is the most potent in lowering serum IL-4 level. One way ANOVA (Table 5) indicated that the general effect on serum TNF-α and IL-4 levels between groups was very highly significantly (P < 0.001, F-probability) throughout the experiment. Two-way ANOVA (Table 6) revealed that the dose effect of latex and extract was very highly significant (P < 0.001; F-probability). The dose effect of ABM was highly significant (P < 0.01; F-probability) on IL-4 level while it was very highly significant (P < 0.001; F-probability) on TNF-α level. The time and dose–time interaction of extract and ABM had a highly significant effect (P < 0.01; F-probability) effect on IL-4 level. However, while time of administration of latex had significant effect (P < 0.05; F-probability) on IL-4 level, its interaction with dose had insignificant effect (P > 0.05; F-probability). The effect of time of ABM on TNF-α level was significant (P < 0.05; F-probability) while its interaction with dose was insignificant (P > 0.05; F-probability).

**Table 5:** Effect of latex and ethanolic leaf extract and abamectin on TNF-α and IL-4 levels in serum of normal rats

Treatment	Parameter			
	TNF-α (pg/ml)		IL-4 (ng/ml)	
	4 weeks	8 weeks	4 weeks	8 weeks
Vehicle (CMC 1 %) control	36.72 ± 1.02^h^	38.72 ± 0.62^h^	202 ± 4.37^a^	197 ± 1.64^a^
1/20 LD_50_ latex	78.55 ± 6.28^fg^	72.55 ± 7.10^g^	130.7 ± 13.63^c^	170.5 ± 15.17^b^
1/10 LD_50_ latex	91.43 ± 6.12^def^	80.30 ± 5.74^efg^	101.53 ± 3.99^d^	112.9 ± 5.34^cd^
1/20 LD_50_ ethanolic extract	102.42 ± 6.53^cd^	92.40 ± 3.67^de^	179.3 ± 7.96^ab^	186.97 ± 4.27^ab^
1/10 LD_50_ ethanolic extract	109.85 ± 4.29^bc^	100.98 ± 3.52^cd^	138 ± 11.67^c^	181.4 ± 3.96^ab^
1/20 LD_50_ abamectin	100.67 ± 5.61^cd^	86.62 ± 3.83^ef^	184.9 ± 8.89^ab^	199.27 ± 2.80^a^
1/10 LD_50_ abamectin	124 ± 3.08^a^	117.95 ± 1.08^ab^	134.97 ± 19.22^c^	197.23 ± 1.95^a^
F-probability	P < 0.001		P < 0.001	
LSD at 5 % level	13.4927		26.4573	
LSD at 1 % level	18.1709		35.6306	

Data are expressed as mean ± SE

Number of animals in each group is six

For each parameter, means, which do not share the same superscript symbol(s), are significantly different at P < 0.05

**Table 6:** Analysis of variance for concentrations in serum on TNF-α and IL-4 levels of normal and treated rats

Source of variation	F-probability	
	TNF-α (pg/ml)	IL-4 (ng/ml)
A—Normal–latex effect		
Dose	P < 0.001	P < 0.001
Time	P > 0.05	P < 0.05
Dose–time	P > 0.05	P > 0.05
B—Normal–extract effect		
Dose	P < 0.001	P < 0.001
Time	P > 0.05	P < 0.01
Dose–time	P > 0.05	P < 0.01
C—Normal–abamectin effect		
Dose	P < 0.001	P < 0.01
Time	P < 0.05	P < 0.01
Dose–time	P > 0.05	P < 0.01

### Effect on heart and testis oxidative stress and antioxidant markers levels

The effects of latex, ethanolic extract of leaves of *C*. *procera* and ABM on GSH, LPO concentrations and GPx, GST and SOD activities are expressed in Tables 7, 8, 9 and 10. GSH content in heart and testis was highly significantly decreased (P < 0.01; LSD) after administration of the plant latex and extract as well as ABM at 4th and 8th week. With the exception of 1/20 LD_50_ of latex on testis LPO, the two tested doses of latex, ethanolic extract and ABM induced a significant elevation of LPO in heart and testis at the 2 tested periods. Heart GPx, GST and SOD activities were detectably decreased after administration of the tested materials at the two tested periods. With the exception of the effect of 1/20 ABM on GPx activity, the GPx, GST and SOD activities were significantly decreased (P < 0.01; LSD) after all treatments at the 4th and 8th week as a result of both tested doses. Regarding one way ANOVA (Tables 8, 9), it was found that the general effect on heart and testis GSH content, LPO and the activities of GPx, GST and SOD between groups was very highly significantly (P < 0.001, F-probability) throughout the experiment.

**Table 7:** Effect of latex and ethanolic leaf extract and abamectin on cardiac GSH content, LPO and GPx, GST and SOD activities in normal rats

Treatments	Parameter									
	GSH (nmol/100 mg tissue)		LPO (nmol/100 mg tissue)		GPx (U/g tissue)		GST (U/g tissue)		SOD (U/g tissue)	
	4 weeks	8 weeks	4 weeks	8 weeks	4 weeks	8 weeks	4 weeks	8 weeks	4 week	8 weeks
Vehicle (CMC 1 %) control	163.34 ± 1.44^a^	143.42 ± 0.93^b^	46.31 ± 0.71^f^	47.85 ± 2.11^f^	84.85 ± 0.99^a^	85.50 ± 2.25^a^	489.93 ± 14.33^ab^	506.25 ± 3.74^a^	14.35 ± 0.18^bc^	15.31 ± 0.71^ab^
1/20 LD_50_ latex	109.87 ± 7.52^cd^	119.65 ± 8.74^c^	64.50 ± 3.68^de^	61.84 ± 1.13^e^	77.39 ± 2.04^bc^	69.00 ± 1.24^defg^	447.92 ± 2.69^ef^	481.80 ± 2.60^bc^	9.07 ± 0.23^e^	11.95 ± 0.29^d^
1/10 LD_50_ latex	69.69 ± 6.29^e^	97.71 ± 6.10^d^	72.82 ± 2.17^cd^	67.16 ± 2.65^cde^	69.93 ± 2.70^defg^	63.96 ± 0.43^g^	429.86 ± 12.39^f^	460.07 ± 6.63^cde^	1.93 ± 0.23^h^	4.62 ± 0.30^g^
1/20 LD_50_ ethanolic extract	68.39 ± 6.23^e^	108.40 ± 1.00^cd^	65.19 ± 3.75^de^	59.78 ± 4.33^e^	68.06 ± 1.69^efg^	79.63 ± 1.3^ab^	480.49 ± 13.70^bc^	454.51 ± 8.65^de^	15.71 ± 0.14^a^	6.84 ± 0.78^f^
1/10 LD_50_ ethanolic extract	45.35 ± 5.28^f^	102.50 ± 8.26^cd^	84.53 ± 4.66^a^	72.71 ± 2.56^cd^	63.96 ± 2.96^g^	77.11 ± 0.74^bc^	472.31 ± 5.63^bcd^	386.06 ± 8.17^h^	2.11 ± 0.25^h^	4.04 ± 0.06^g^
1/20 LD_50_ ABM	118.73 ± 11.35^c^	116.51 ± 4.20^c^	76.55 ± 6.46^abc^	72.56 ± 2.02^cd^	74.22 ± 2.59^bcd^	72.35 ± 3.33^cdef^	470.49 ± 3.39^bcde^	487.85 ± 0.77^ab^	13.39 ± 0.11^c^	7.12 ± 0.15^f^
1/10 LD_50_ ABM	77.61 ± 0.70^e^	77.43 ± 1.31^e^	84.30 ± 5.44^ab^	74.19 ± 3.12^bcd^	72.54 ± 2.35^cde^	84.85 ± 0.99^a^	447.92 ± 7.12^ef^	395.83 ± 3.36^g^	12.26 ± 0.22^d^	4.84 ± 0.38^g^
F-probability	P < 0.001		P < 0.001		P < 0.001		P < 0.001		P < 0.001	
LSD at 5 % level	17.2065		10.2926		6.0102		22.709		1.013	
LSD at 1 % level	23.1723		13.8613		8.0941		30.5827		1.3642	

Data are expressed as mean ± SE

Number of animals in each group is six

For each parameter, means, which do not share the same superscript symbol(s), are significantly different at P < 0.05

**Table 8:** Effect of latex and ethanolic leaf extract and abamectin on testicular GSH content, LPO and GPx, GST and SOD activities in normal rats

Treatments	Parameter									
	GSH (nmol/100 mg tissue)		LPO (nmol/100 mg tissue)		GPx (U/g tissue)		GST (U/g tissue)		SOD (U/g tissue)	
	4 weeks	8 weeks	4 weeks	8 weeks	4 weeks	8 weeks	4 weeks	8 weeks	4 week	8 weeks
Vehicle (CMC 1 %) control	86.78 ± 9.11^a^	69.60 ± 6.12^b^	13.68 ± 1.72^e^	14.46 ± 2.40^e^	71.89 ± 3.25^a^	73.20 ± 6.68^a^	976.18 ± 23.75^a^	1011.18 ± 27.05^a^	10.24 ± 1.77^a^	9.33 ± 0.97^ab^
1/20 LD_50_ latex	68.97 ± 3.94^b^	33.41 ± 5.68^e^	16.30 ± 0.56^de^	23.33 ± 1.97^bc^	50.85 ± 6.52^cd^	44.53 ± 3.42^cde^	683.16 ± 37.74^bcd^	787.50 ± 52.34^b^	6.45 ± 0.38^cde^	5.12 ± 0.44^defg^
1/10 LD_50_ latex	54.24 ± 1.85^cd^	28.32 ± 3.02^ef^	29.76 ± 1.98^a^	30.06 ± 3.02^a^	48.50 ± 4.38^cde^	46.99 ± 4.29^cde^	637.50 ± 42.81^cde^	769.38 ± 29.28^b^	5.53 ± 0.54^def^	4.40 ± 0.24^efg^
1/20 LD_50_ ethanolic extract	64.06 ± 2.18^bc^	19.03 ± 0.55^fg^	25.37 ± 2.67^abc^	21.35 ± 1.73^cd^	55.13 ± 3.69^bc^	49.06 ± 1.37^cd^	753.96 ± 33.91^bc^	673.09 ± 76.22^bcd^	7.93 ± 0.98^bc^	6.36 ± 0.56^cde^
1/10 LD_50_ ethanolic extract	48.05 ± 1.77^d^	14.87 ± 0.59^g^	28.10 ± 2.42^ab^	22.56 ± 1.65^bc^	49.44 ± 2.15^cd^	37.27 ± 3.28^e^	489.58 ± 6.99^f^	584.21 ± 47.88^def^	7.13 ± 0.46^cd^	3.07 ± 0.76^g^
1/20 LD_50_ ABM	53.45 ± 4.58^cd^	31.06 ± 3.74^e^	21.72 ± 1.61^cd^	21.80 ± 1.50^cd^	64.25 ± 3.05^ab^	51.47 ± 2.67^cd^	672.29 ± 21.79^bcd^	596.88 ± 66.45^def^	6.99 ± 0.80^cd^	4.05 ± 0.37^fg^
1/10 LD_50_ ABM	48.87 ± 3.79^d^	18.42 ± 0.96^fg^	23.33 ± 0.87^bc^	27.92 ± 1.99^ab^	52.10 ± 3.18^cd^	41.90 ± 3.22^de^	569.17 ± 29.95^def^	546.87 ± 51.57^ef^	5.06 ± 0.48d^efg^	3.59 ± 0.40^fg^
F-probability	P < 0.001		P < 0.001		P < 0.001		P < 0.001		P < 0.001	
LSD at 5 % level	11.946		5.6821		11.3121		124.2059		2.1784	
LSD at 1 % level	16.0879		7.6522		15.2342		167.2704		2.9337	

Data are expressed as mean ± SE

Number of animals in each group is six

For each parameter, means, which do not share the same superscript symbol(s), are significantly different at P < 0.05

**Table 9:** Analysis of variance for oxidative stress and antioxidant enzymes in heart of normal and treated rats

Source of variation	F-probability				
	GSH	LPO	GPx	GST	SOD
A—Normal–latex effect					
Dose	P < 0.001	P < 0.001	P < 0.001	P < 0.001	P < 0.001
Time	P > 0.05	P > 0.05	P < 0.01	P < 0.001	P < 0.001
Dose–time	P < 0.01	P > 0.05	P < 0.05	P > 0.05	P < 0.05
B—Normal–extract effect					
Dose	P < 0.001	P < 0.001	P < 0.001	P < 0.001	P < 0.001
Time	P < 0.001	P > 0.05	P < 0.001	P < 0.001	P < 0.001
Dose–time	P < 0.001	P > 0.05	P < 0.01	P < 0.001	P < 0.001
C—Normal–abamectin effect					
Dose	P < 0.001	P < 0.001	P < 0.001	P < 0.001	P < 0.001
Time	P > 0.05	P > 0.05	P > 0.05	P > 0.05	P < 0.001
Dose–time	P > 0.05	P > 0.05	P > 0.05	P < 0.001	P < 0.001

**Table 10:** Analysis of variance for oxidative stress and antioxidant markers in testis of normal and treated rats

Source of variation	F-probability				
	GSH	LPO	GPx	GST	SOD
A—Normal–latex effect					
Dose	P < 0.001	P < 0.001	P < 0.001	P < 0.001	P < 0.001
Time	P < 0.001	P > 0.05	P > 0.05	P < 0.01	P > 0.05
Dose–time	P > 0.05	P > 0.05	P > 0.05	P > 0.05	P > 0.05
B—Normal–extract effect					
Dose	P < 0.001	P < 0.001	P < 0.001	P < 0.001	P < 0.001
Time	P < 0.001	P > 0.05	P > 0.05	P > 0.05	P < 0.05
Dose–time	P < 0.05	P > 0.05	P > 0.05	P > 0.05	P > 0.05
C—Normal–abamectin effect					
Dose	P < 0.001	P < 0.001	P < 0.001	P < 0.001	P < 0.001
Time	P < 0.001	P > 0.05	P < 0.05	P > 0.05	P < 0.05
Dose–time	P > 0.05	P > 0.05	P > 0.05	P > 0.05	P > 0.05

Concerning two-way ANOVA (Tables 9, 10), the dose effect of latex, ethanolic extract and ABM on GSH content and GPx, GST and SOD activities in heart and testis was very highly significant (P < 0.001, F-probability) throughout the experiment. Regarding latex effect, the time had insignificant effect (P > 0.05, F-probability) on heart GSH content and LPO, testis LPO, testis GPx and SOD activities and highly significantly effect (P < 0.01, F-probability) on heart GPx and testis GST activities and very highly significantly effect (P < 0.001, F-probability) on testis GSH level and activities of heart SOD and GST. The effect of interaction between dose and time was highly significant (P < 0.01, F-probability) on heart GSH content and only significant (P < 0.05, F-probability) on heart GPx and SOD activities. Concerning extract effect, the time had very highly significant effect (P < 0.001, F-probability) on cardiac GPx, GST and SOD activities and cardiac and testicular GSH levels and only significant effect on testicular SOD activity. Dose–time interaction had a very highly significant effect (P < 0.001, F-probability) on cardiac GSH content and GST and SOD activities and a highly significant effect on GPx activity. Concerning to ABM, the time had a significant effect (P < 0.05, F-probability) on testicular GPx and SOD activities and a very highly significant effect (P < 0.001, F-probability) on cardiac SOD activity and testicular GSH level. Dose–time interaction had a very highly significant effect (P < 0.001, F-probability) on cardiac GST and SOD activities.

### Histopathological effects

**Fig. 1:**
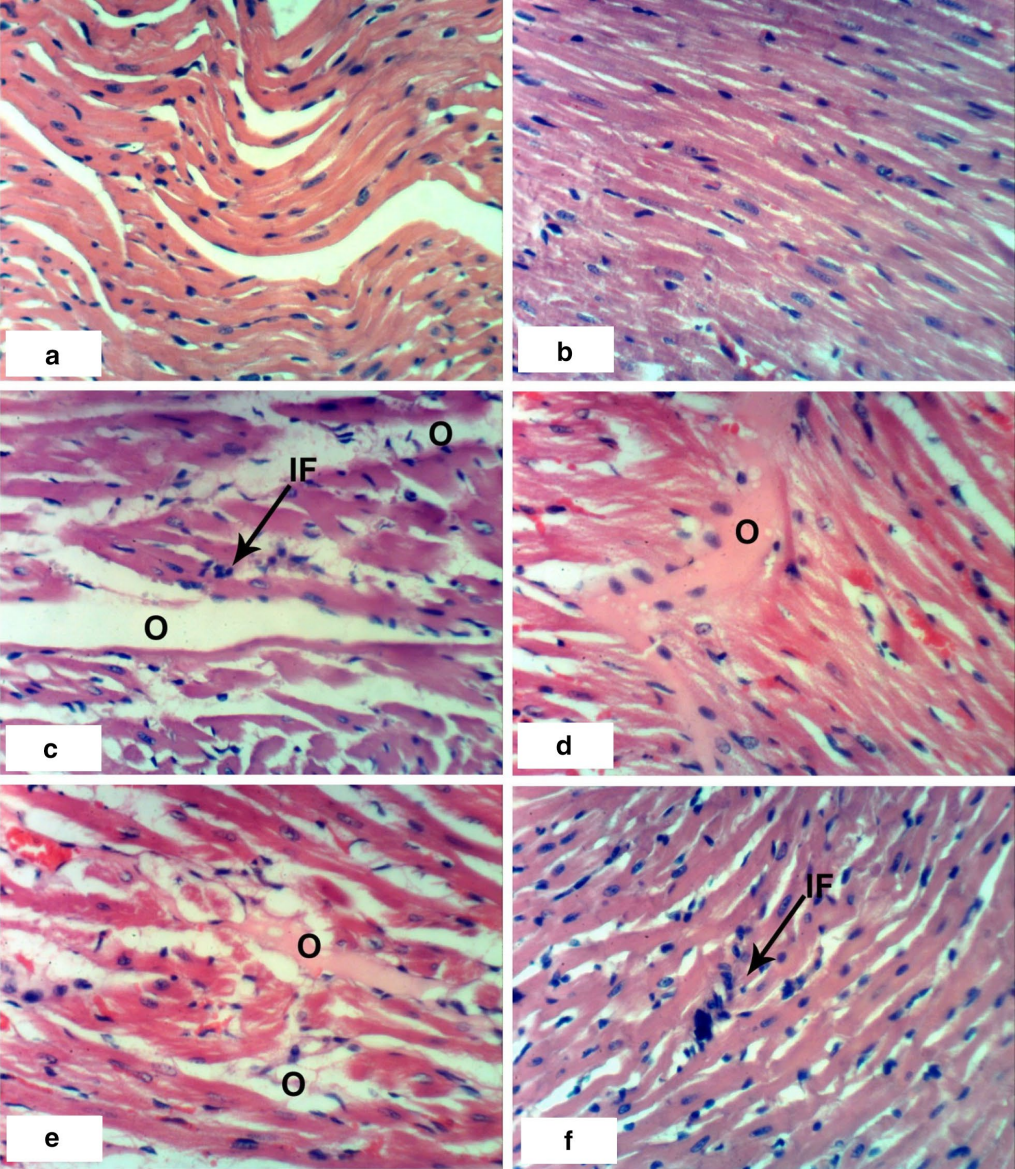
Photomicrographs of H and E stained heart sections of normal and latex treated rats. Sections of control rats administered 1 % CMC for 4 weeks (**a**) and 8 weeks (**b**) showing normal myocytes of heart. **c** Section of rat treated with 1/20 LD_50_ of latex for 4 weeks showing intermuscular odema (O) associated with inflammatory cell infiltration (IF), **d**, **e** sections of rats treated with 1/20 LD_50_ of latex for 8 weeks showing intermuscular odema (O), **f** section of rat treated with 1/10 LD_50_ of latex for 4 weeks showing inflammatory cell infiltration (IF) (×400)

**Fig. 2:**
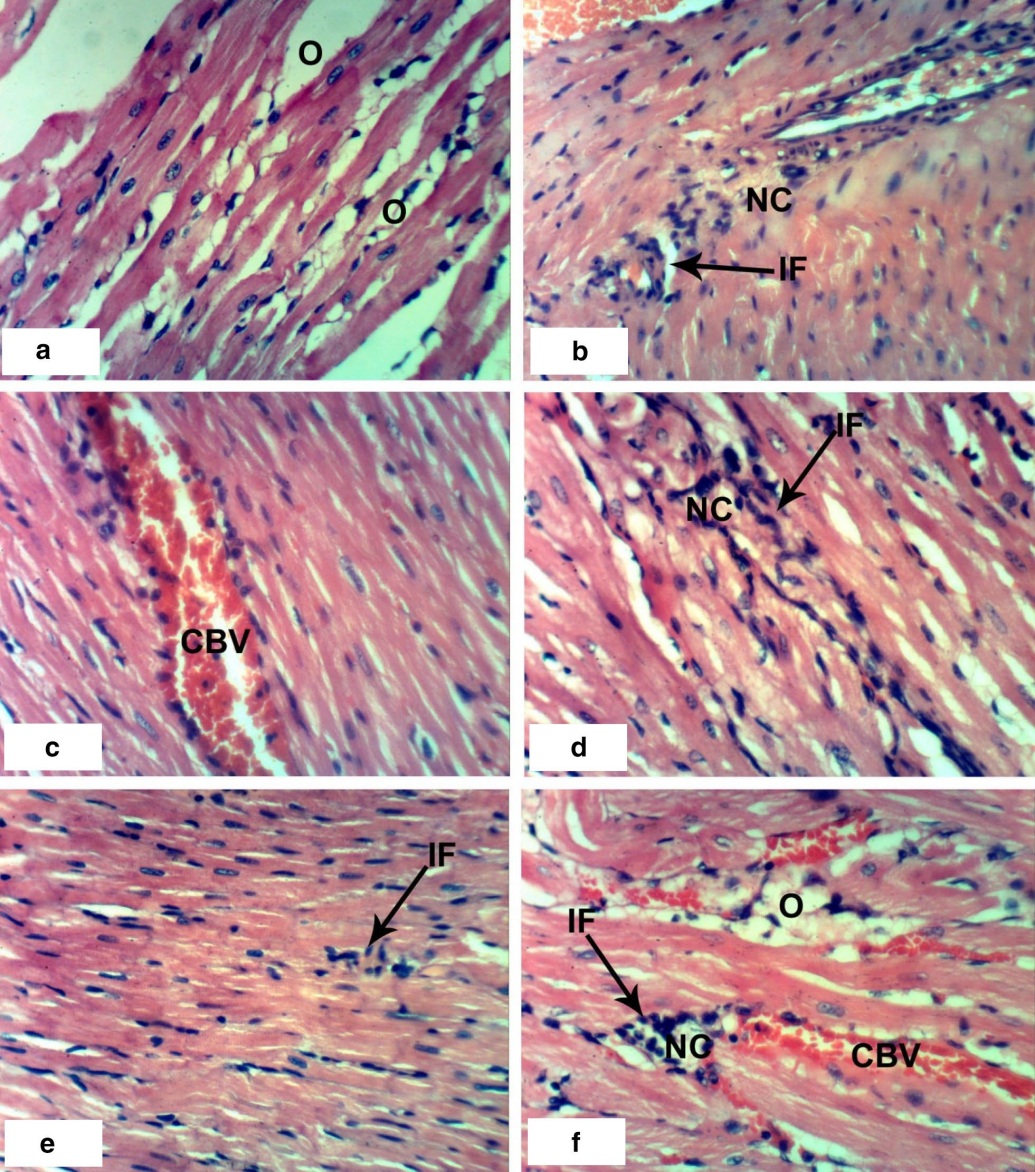
Photomicrographs of H and E stained heart sections of latex and extract treated rats. **a** Section of rat treated with 1/10 LD_50_ of latex for 4 weeks showing intermascular odema (O), **b**–**d** sections of rats treated with 1/10 LD_50_ of latex for 8 weeks showing congestion of blood vessels (CBV) and necrosis (NC) associated with inflammatory cell infiltration (IF), **e** SECTION of rat treated with 1/20 LD_50_ of extract for 4 weeks showing inflammatory cell infiltration (IF), **f** section of rat treated with 1/20 LD_50_ of extract for 8 weeks showing intermuscular odema (O), necrosis (NC) and congestion of blood vessels (CBV) (×400)

**Fig. 3:**
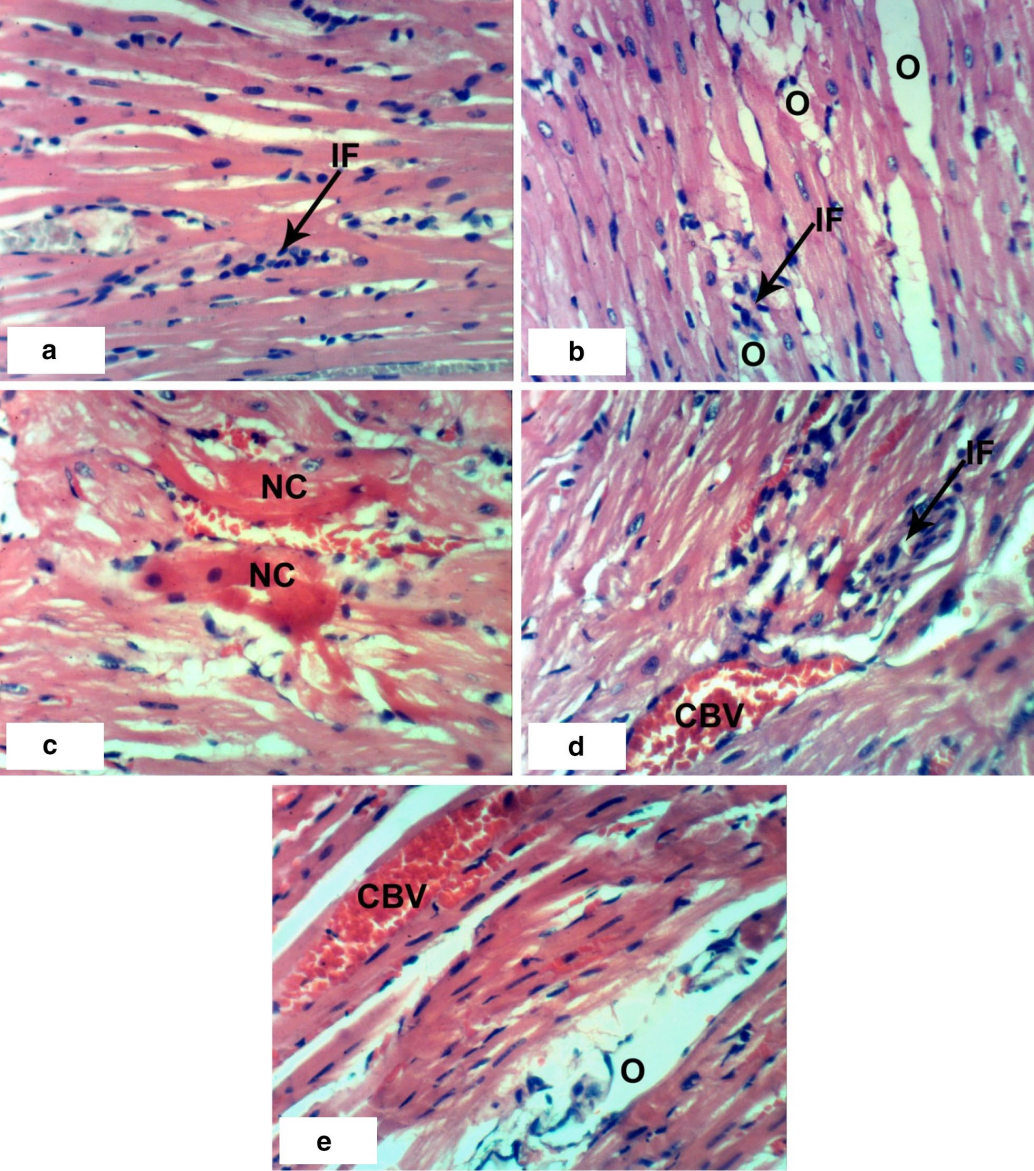
Photomicrographs of H and E stained heart sections of rats treated with extract. **a**, **b** Sections of rats treated with 1/10 LD_50_ extract for 4 weeks showing odema and inflammatory cell infiltration (IF), **c**–**e** sections of rats treated with 1/10 LD_50_ of extract for 8 weeks showing necrosis (NC), congestion of blood vessels (CBV), inflammatory cell infiltration (IF) and odema (O) (×400)

**Fig. 4:**
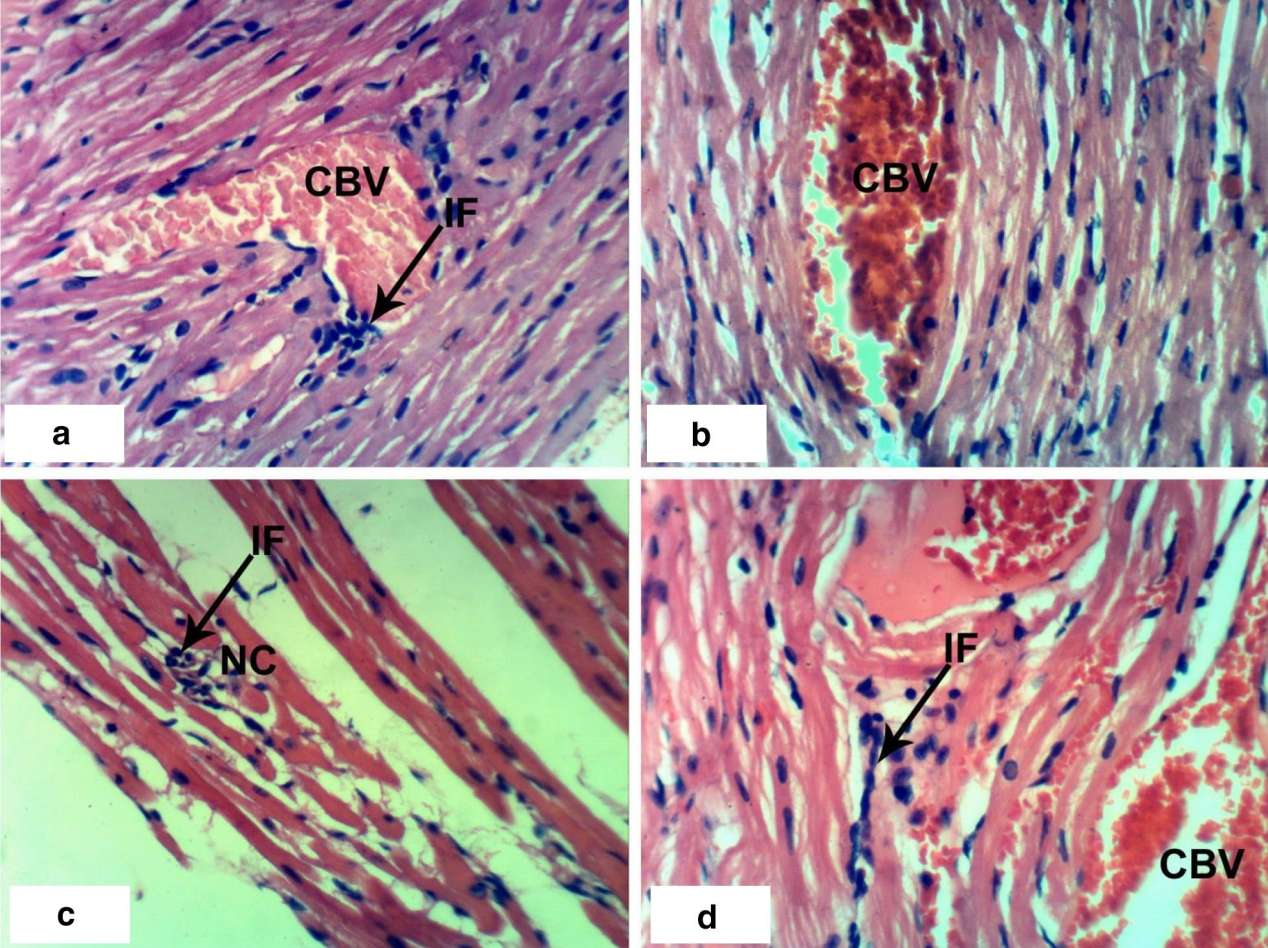
Photomicrographs of H and E stained heart sections of ABM treated rats. **a** Section rat treated with 1/20 LD_50_ ABM for 4 weeks showing inflammatory cell infiltration (IF) and congestion of blood vessels (CBV), **b** section of rat treated with 1/20 LD_50_ of ABM for 8 weeks showing congestion of blood vessels (CBV), **c** section of rat treated with 1/10 LD_50_ ABM for 4 weeks showing necrosis (NC) associated with inflammatory cell infiltration (IF), **d** section of rat treated with 1/10 LD_50_ ABM for 8 weeks showing congestion of blood vessels (CBV) and inflammatory cell infiltration (IF) (×400)

Normal myocytes architecture of normal rat heart in control animals were observed (Fig. 1a, b). Treatments of normal rats with 1/20 of LD_50_ of *C. procera* latex caused intermuscular odema (Fig. 1c–e) associated with inflammatory cell infiltration (Fig. 1c). Administration of 1/10 of LD_50_ of latex caused marked alterations of normal structure of heart by affecting on cardiac myocytes causing odema and inflammatory cell infiltration (Figs. 1f, 2a) in short time (4 weeks) while after 8 weeks, it caused necrosis associated with inflammatory cells infiltration and congestion of blood vessels (Fig. 2b–d). The treatment of rats with 1/20 of LD_50_ of ethanolic extract caused inflammatory cells infiltration at the end of the 4th week (Fig. 2e) and it caused intermuscular odema, necrosis of cardiac myocytes associated with inflammatory cells infiltration and congestion of blood vessels at the end of the 8th week (Fig. 2f). Increasing the concentration of the ethanolic extract (1/10 of LD_50_) caused odema associated with inflammatory cell infiltration (Fig. 3a, b) after 4 week. Prolongation of period of administration of this dose caused congestion of blood vessels, necrosis of cardiac myocytes associated with inflammatory cell infiltration (Fig. 3c–e). Regarding ABM administration, low dose caused congestion of blood vessels and inflammatory cells infiltration (Fig. 4a, b) and high dose caused necrosis of cardiac myocytes associated with inflammatory cell infiltration (Fig. 4c, d).

**Fig. 5:**
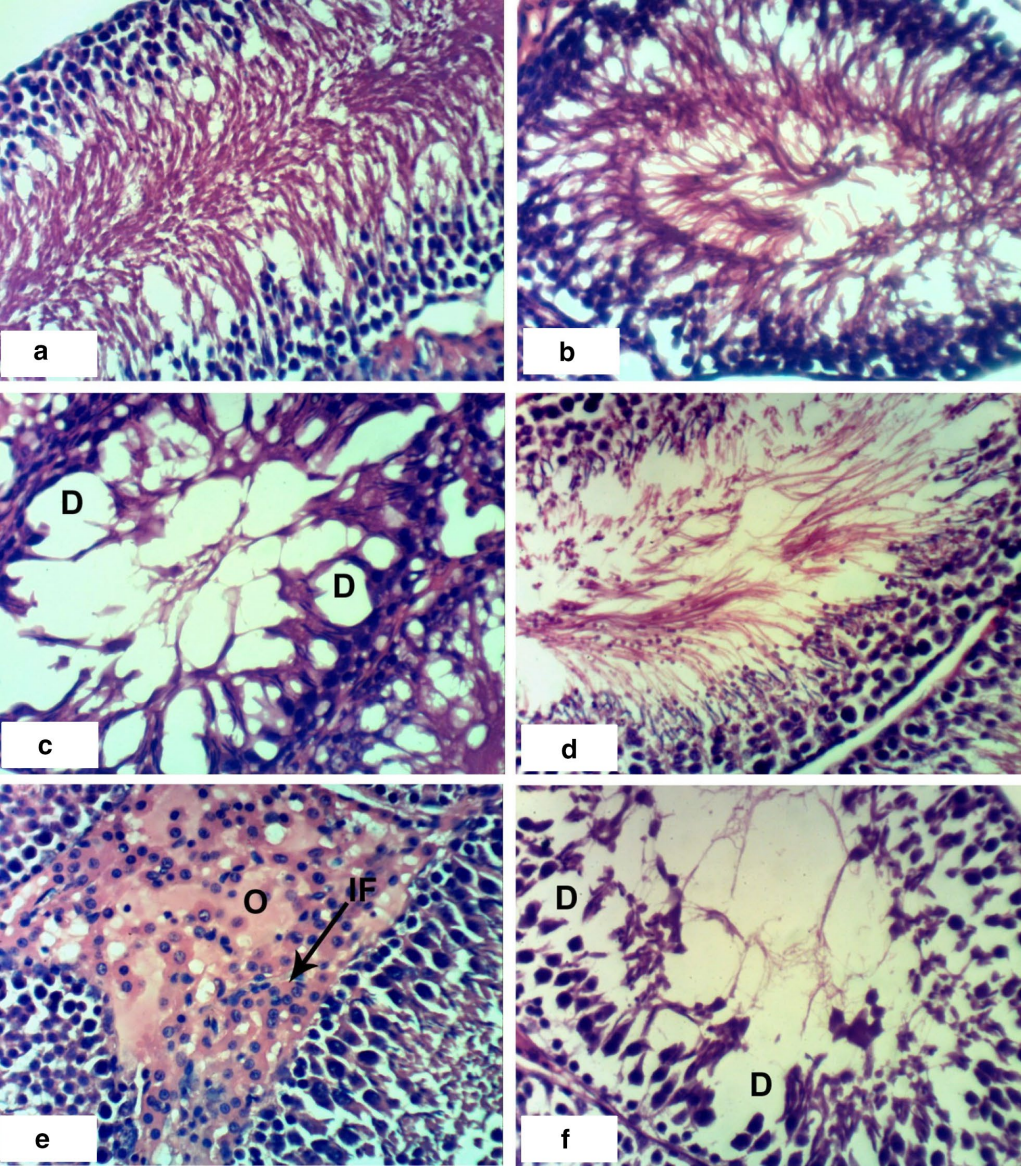
Photomicrographs of H and E stained testis sections of control and latex treated rats. Sections of control rats administered 1 % CMC for 4 weeks (**a**) and for 8 weeks (**b**) showing normal histologic structure of testis and successive stages of spermatogenesis, **c** section of rat treated with 1/20 LD_50_ latex for 4 weeks showing degeneration of spermatogonial cells (D), **d** section of rat treated with 1/20 LD_50_ latex for 8 weeks showing mild degeneration of spermatids (D), **e** section of rat treated with 1/10 LD_50_ latex for 4 weeks showing odema (O) associated with inflammatory cell infiltration (IF), **f** section of rat treated with 1/10 LD_50_ latex for 8 weeks showing degeneration of spermatogonial cells (D) (×400)

**Fig. 6:**
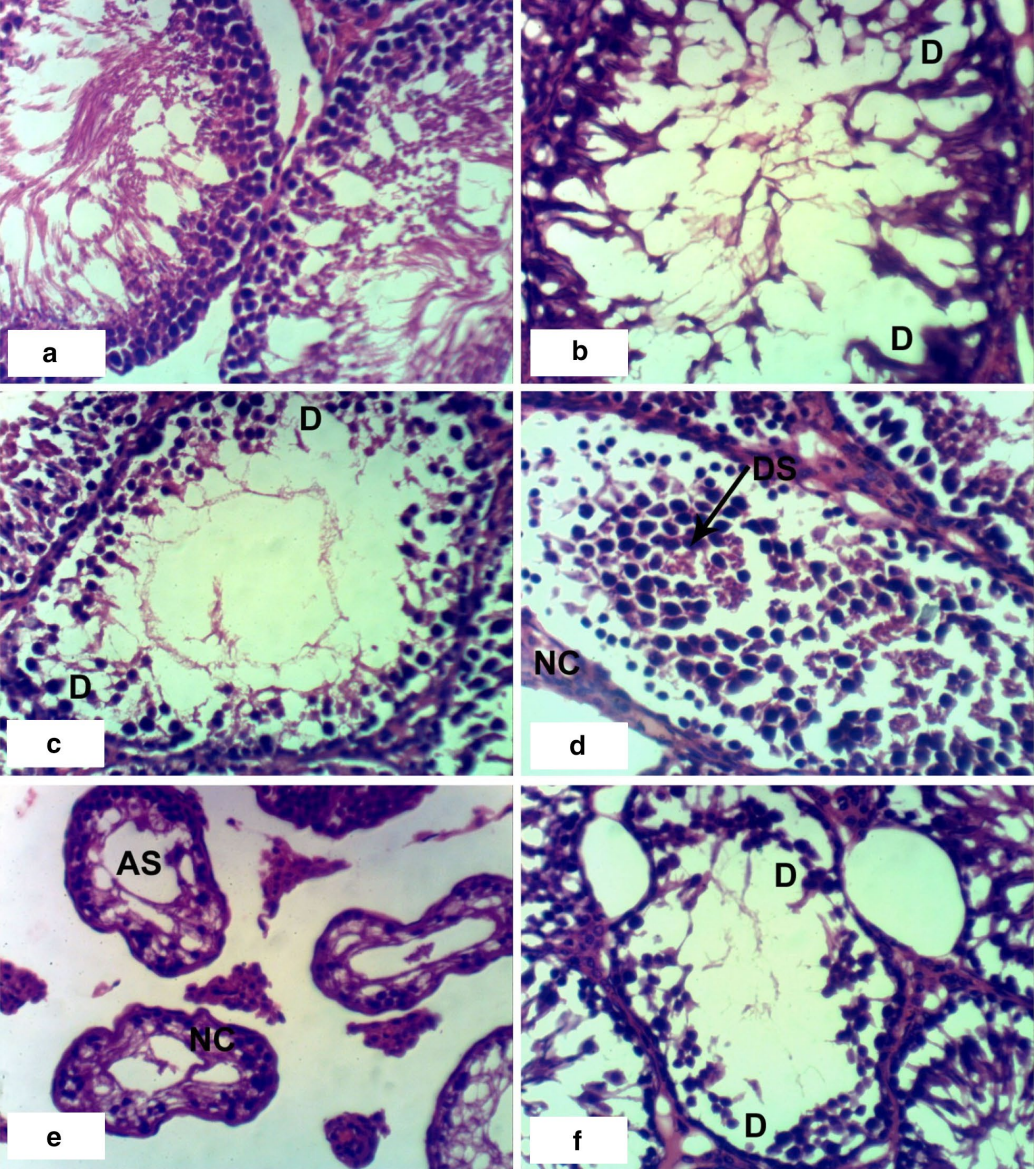
Photomicrographs of H and E stained testis sections of extract treated rats. **a** Section of rat treated with 1/20 LD_50_ extract for 4 weeks showing normal structure of testis, **b** section of rat treated with 1/20 LD_50_ extract for 8 weeks showing degeneration of spermatogonial cells (D), **c** section of rat treated with 1/10 LD50 extract for 4 weeks showing degeneration of spermatogonial cells (D), **d**–**f** sections of rats treated with 1/10 LD_50_ extract for 8 weeks showing desquamation of cells (DS), necrosis (NC), atrophy of seminiferous tubules (AS) and degeneration of spermatogonial cells (D) (×400)

**Fig. 7:**
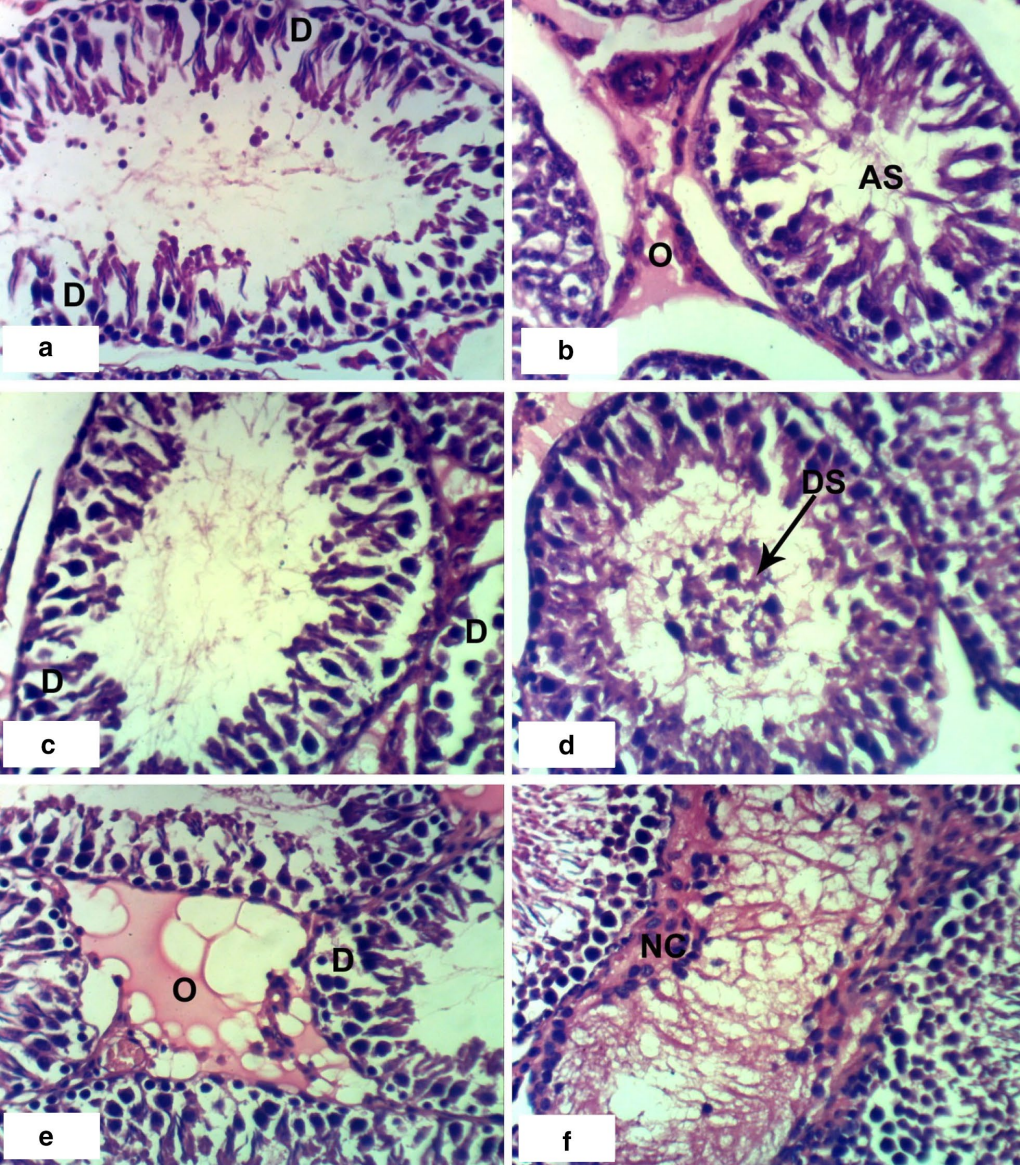
Photomicrographs of H and E stained testis sections of ABM treated rats. **a** Section of rat treated with 1/20 LD_50_ ABM for 4 weeks showing degeneration of spermatogonial cells (D), **b**–**d** sections of rats treated with 1/20 LD_50_ ABM for 8 weeks showing atrophy of seminiferous tubules (AS), odema (O) degeneration of spermatogonial cells (D) and desquamation of cells (DS), **e**, **f** sections of rats treated with 1/10 LD_50_ ABM for 4 weeks showing degeneration of spermatogonial cells (D), odema (O) and necrosis of cells (NC) (×400)

**Fig. 8:**
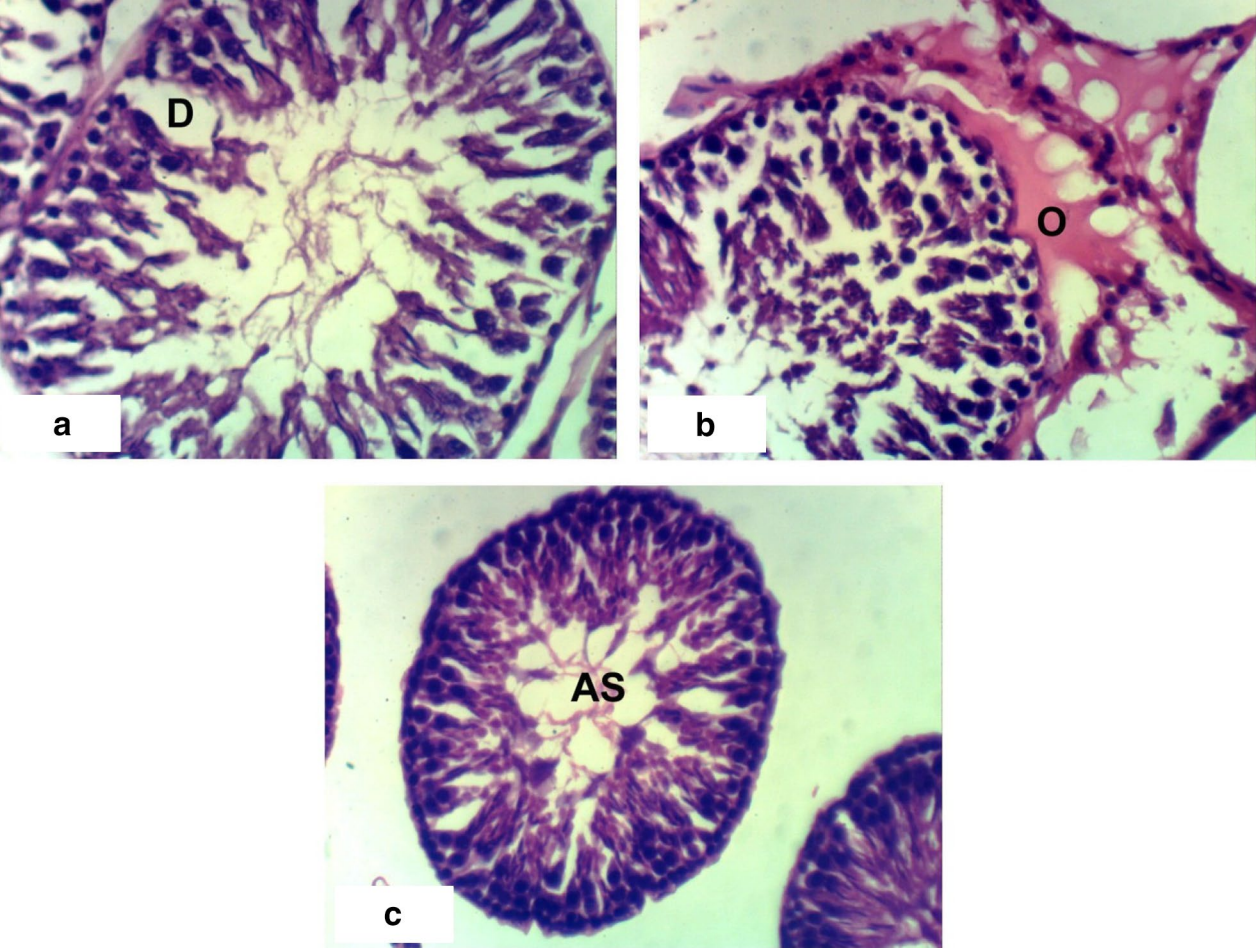
Photomicrographs of H and E stained testis sections of ABM treated rats for 8 weeks showing degeneration of spermatogonial cells (D) (photomicrograph **a**), odema (O) (photomicrograph **b**) and atrophy of seminiferous tubules (AS) (photomicrograph **c**) (×400)

Control groups demonstrated normal testicular histology with all successive stages of spermatogenesis (Fig. 5a, b). Administration of 1/20 LD_50_ latex for 4 weeks altered the normal testis structure by causing degeneration of spermatogonial cells lining seminiferous tubules (Fig. 5c). Interstitial oedema associated with inflammatory cells infiltration (Fig. 5e) occurred after administration of the high dose of latex after 4 weeks and degeneration observed after 8 weeks (Fig. 5f). Administration of 1/10 LD_50_ ethanolic extract of *C*. *procera* for 8 weeks caused atrophy of the seminiferous tubules, necrosis, degeneration and desquamation of spermatogonial cells lining seminiferous tubules (Fig. 6d–f). Treatment with ABM at the low dose caused degeneration and atrophy of the seminal vesicles and desquamation of spermatogonial cells (Fig. 7b–d). Treatment with the high dose of ABM for 4 weeks caused degeneration and interstitial odema and necrosis (Fig. 7e, f). After 8 weeks administration, appearance of degeneration, interstitial odema and atrophy of seminal vesicles (Fig. 8a–c) were observed.

## Discussion

Plant extracts may provide an alternative method to currently applied pesticides, as they constitute a rich source of bioactive chemicals (Kim et al. 2005).

*Calotropis procera* plant commonly called Sodom apple or Giant milkweed belong to the family of Asclepiadaceae. It is a major grazing plant found in Asian temperate region, Asia-tropical and Africa (Agaie et al. 2007). It was reported that ingestion of fresh *C. procera* leaves and latex has been suggested as toxic to many ruminants by several farmers (Mahmoud et al. 1979a; Singhal and Kumar 2009). It was reported by Thankamma (2003) and Basak et al. (2009) that *C. procera* latex administered to rats revealed toxic, wound healing, and pain-killing effects. Chemical compounds in the latex are calotropagenin glycosides/derivatives, cardenolides, flavonoids, and saponins (Kanojiya and Madhusudanan 2012). Cardenolides in the *C. procera* latex are associated with the toxic effects in mammals (Elgamal et al. 1999). Phytochemical screening of the extracts of *C. procera* leaves indicated the presence of alkaloids, carbohydrates, cardiac glycosides, saponins, phenols, tannins, terpenoids and flavanoids which are known to possess medicinal and pesticidal properties (Verma et al. 2013). It was reported by De Lima et al. (2011) that the plant as hepatotoxic and cardiotoxic. Other researchers have documented the renal toxicity in addition to hepatic toxicity of the plant (Basak et al. 2009; Lin and Will 2012). The chemical poisons from plants such as Argel (*Solenostemma argel*) and Usher (*C. procera*) are mostly alkaloids which are nitrogenous heterocyclic compounds having strong effects on the nervous system of animals and may result in death (Badshah et al. 2004).

In view of this study, administration of *C*. *procera* latex and ethanolic extract of leaves for 4 and 8 weeks induced significant elevations in the activities of CK-MB, AST and LDH. These results are in accordance with those of El Badwi and Bakhiet (2010) and El-Badwi et al. (2010).

In the current study, histological examination of rats treated with *C*. *procera* latex and ethanolic leaf extract showed impairment of the normal structure of heart. Early histopathological changes at the 4th week of latex and extract administration include intermuscular odema, inflammatory cell infiltration. As the period of latex and extract administration extended to 8 weeks, alterations are more pronounced and include necrosis of cardiac myocytes associated with inflammatory cells infiltration and congestion of blood vessels. These results go parallel with these of El Badwi and Bakhiet (2010) who reported that the heart muscle fibers were focally vacuolated or necrotic with lymphatic infiltration. It was reported by De lima et al. (2011) that the toxic effects, established by intraperitoneal injection of *C. procera* latex to rats and oral administration of chopped leaves in a with the lowest amount of water to sheep appeared as cardiac muscle fibers separated by edematous fluid, and the rats exhibited subendocardic hemorrhages, infiltration of mononuclear inflammatory cells, multi-focal coagulation necrosis of the muscular fibers evidenced by granular appearance of the sarcoplasm, distinct eosinophilic cytoplasm lacking transverse striations, basophilic granulation and prominent vacuolization of the sarcoplasm of some fibres and presenting pyknotic or absent nuclei.

Phytochemical studies have revealed that *C. procera* contains a mixture of cardenolides, including proceragenin and 2″-oxovoruscharin (Van Quaquebeke et al. 2005). Cardenolides are cardiac-active compounds that inhibit the cellular membrane Na^+^/K^+^ ATPase, resulting in an electrolytic disturbance that affects the electrical conductivity of the heart (Poindexter et al. 2007). Thus, the heart dysfunction and the elevations in heart enzymes (CK-MB, AST and LDH) in serum and cardiac toxicity induced by the plant, in this study, may attributed to its constituting cardenolides. The milky latex contains a powerful bacteriolytic enzyme, toxic glycoside calactin, calotropin D1, calotropin D2 calotropin F11, and a nontoxic powerful proteolytic enzyme and it exhibited local anesthetic activity (Samar et al. 2009). In this study, administration of ABM showed marked elevations in LDH, AST and CK-MB. These results reflected the toxic effect of ABM and impairment of heart function. The elevated of serum enzymes related to heart function was associated with cardiac histopathological lesions which include oedema, inflammatory cells infiltration and necrosis observed in the present investigation.

In view of this study, treatments with *C. procera* latex and ethanolic extract of leaves induced marked decrease in the levels of male sex hormones testosterone, FSH and LH. In conduction with the present study, Sharma and Jacob (2001) found that intermuscular administration of aqueous and ethanolic extracts of flowers of *C. procera* has been shown to induce functional sterility and has a potent antispermatogenic activity in the albino mouse, but at the doses and experimental regimen employed, had no apparent effect on sexual behavior or libido. In the same way, Akinloye et al. (2002) reported that fresh leaves extract has depicted potential deleterious effects on the rat testes and accessory sex organs represented by degeneration of seminiferous epithelium of varying degrees as well as presence of large-sized multimate cells in the tubules and empty interstitial spaces. *Calotropis* is extensively used in both male and female rats for understanding its role in fertility (Akinloye et al. 2002; Circosta et al. 2001; Ahirwar et al. 2007). It was indicated previously that, an active principle of flower extract of *C. procera* showed spermicidal effect on testicular functions in Indian desert male Gerbil (Garg 1979).

Histologic profile of testes in the present study revealed extensive deleterious changes in the germinal tubules which contained mainly necrotic and degenerating germ cells. Further, the epididymal lumina appeared devoid of spermatozoa and exhibited mainly cell debris. Also, the seminiferous tubules were atrophied and necrosis and desquamation of spermatogonial cells after administration of the high dose of the extract. The interstitium was observed to be devoid of leydig cells in this study. This change may be due to decreased production of testosterone known to be responsible for normal testicular architecture. Histological changes observed in the testes of treated rats in this study may be due to the cardiac glycosides found in the plant which was incriminated to be responsible also for pathological and ultra-structural changes in the kidney tubules of Wistar rats. These changes are in concordance with Akinloye et al. (2002). In the current study, the plant showed toxic effect on the testis through effect on the germ cell and this is conducted by Akinloye et al. (2002) who said *C. procera* extract has destructive effect on the germ cells which are actively dividing. In addition, it was reported that testosterone maintained the viability of spermatozoa (Bhargava 1989). The toxic effect of the plant in this study to decrease the serum level of testosterone may be mediated via affecting on leydig cells and impairing their functions and structures.

Previous reports have indicated a strong link between male infertility and exposure to more than 50 pesticides (Victor-Costa et al. 2010; Manfo et al. 2010; Tiwari et al. 2011). The adverse effects of ABM on the fertility of adult male rats have been demonstrated in the present study. The serum levels of testosterone, FSH and LH was significantly reduced in rats treated with 1/20 and 1/10 LD_50_ of ABM for 4 and 8 weeks. These results are in agreement with those of Elbetieha and Daas (2003) and Abd-Elhady and Abou-Elghar (2013) who found reduction in testosterone level at dose of 2.13 mg ABM/animal/day. Elbetieha and Daas (2003) indicated that ingestion of ABM for 6 weeks induced adverse effects on male rat fertility and reproduction. The decrease in male fertility via decrease in male sex hormones in rats treated with ABM in the present study is explained by Abd-Elhady and Abou-Elghar (2013) who suggested that ABM may have acted directly on the testes and affected the androgen biosynthesis pathway. These are strongly supported by the wide array of abnormalities seen when histopathological sections of the testes were examined. These abnormalities include necrotic changes in the tissues, interstitial odema, and degeneration and atrophy of seminal vesicles as well as desquamation of spermatogonial cells. In our opinion, it can be suggested that ABM may act directly on the testes and affected the androgen biosynthesis pathway or may directly act on the brain, hypothalamus or anterior pituitary gland which will indirectly affect the testes and possibly affect sexual activity. Both attributions are supported by the present study which revealed direct histological effect of ABM on testis and direct effects on pituitary hormones FSH and LH that respectively control spermatogenesis and testosterone secretion from leydig cells.

The shift in balance between oxidant/antioxidant in favor of oxidants is termed oxidative stress. Oxidative stress contributes to many pathological conditions. When oxidative stress occurs, cells attempt to counteract the oxidant effects and restore the redox balance by activation or silencing of genes encoding defensive enzymes, transcription factors, and structural proteins Scandalios (2004). Glutathione (GSH) is highly abundant in all cell compartments and is the major soluble antioxidant. It detoxifies hydrogen peroxide and lipid peroxides via action of glutathione peroxidase. GSH donates its electron to H_2_O_2_ to reduce it into H_2_O and O_2_. GSH protects cells against apoptosis by interacting with proapoptotic and antiapoptotic signaling pathways (Masella et al. 2005). In this study, it is observed that administration of latex and ethanolic extract induced marked decrease in heart and testis GSH levels and GPx, GST and SOD activities and on the other hand, it induced significant increase in lipid peroxidation. This increase in lipid peroxidation and suppression of antioxidant defense system as a result of latex and ethanolic extract administration lead to excess production and less scavenging of reactive oxygen species which in turn result in cardiac and testicular oxidative damage. In the current study, the deterioration of non-enzymatic and enzymatic antioxidants and exacerbated production of lipid peroxidation was associated with the elevation in the levels of various serum biochemical markers of cardiac and testicular damage including CK-MB, AST and LDH.

Administration of different doses of ABM caused depletion in GSH content, GPx, GST and SOD activities in this study. These results are in line with those of El-Shenawy (2010) who studied the toxic effect of ABM on isolated rat hepatocytes and found ABM decreased GSH concentration and GPx and SOD activities. Since superoxide is the primary ROS produced from the toxic substances, its dismutation by SOD is the primary importance for each cell. So, the depletion of the activity of SOD in this study caused accumulations of ROS in tissues causing disturbance of cell membrane and damage of cells.

Lipid peroxidation is the oxidative deterioration of polyunsaturated lipids to form radical intermediates that bring about cellular damage. Malondialdehyde (MDA), a major end product of this reaction, is an index of lipid peroxidation and has been estimated as thiobarbituric acid (TBARS) (Kohen and Nyska 2002). The increase in MDA level after the latex and ethanolic extract administration reflects that the plant induced increase in ROS and lipid peroxides. The elevation of lipid peroxides caused disturbance in cell membrane structure, damage of cell and cell death. Degree of toxicity induced by the plant is dose dependent. Significant increases in lipid peroxidation in heart and testis after ingestion of ABM in this study were resulted. Increasing dose progressively increased the toxic effect on the normal oxidant/antioxidant state in tissues. These results are in line with those of El-Shenawy (2010) who studied the toxic effect of ABM on isolated rat hepatocytes and found that ABM increased LPO.

Another substantiation of the impairment of the oxidant/antioxidant status in cells occurred with the plant and ABM administration is the depletion of the GPx, GST and SOD activities in tissues in a dose dependent manner. These results are in line with those of El-Shafey et al. (2011) and in contrast with those of El-Shenawy (2010). Based on the findings of the present study, it can be concluded that the cardiotoxic and testicular toxicity of *C. procera* leaf extract and latex as well as ABM is due to the elevation of lipid peroxidation and ROS and depletion of antioxidant levels.

The milky sap is a mixture of various chemicals including calotropis glycosides such as calotropin, calotoxin, calactin, uscharidin, voruscharin which are caustic in nature and are considered poisonous. The irritant and pro-inflammatory property of latex of *C. procera* has been well established (Alencar et al. 2006). Accidental exposure to the latex has been reported to cause inflammation of the skin and eyes (Shivkar and Kumar 2003; Al-Mezaine et al. 2005). In the present study, it was resulted that the latex and ethanolic extract as well as ABM induced marked inflammations as observed in photomicrographs of heart and testis histological sections. This heart and testis inflammatory status was concomitant with the marked elevation of serum levels of pro-inflammatory cytokine, TNF-α, and depletion of anti-inflammatory cytokine, IL-4, in a dose and time dependent manner.

Overall, *C. procera* latex and ethanolic extract of leaves as well as ABM induced cardiotoxic and testicular toxic effects which was evidenced by increases in the activities of heart enzymes in serum and decreases in male sex hormones in serum in addition to heart and testis histological perturbances. However the ethanolic leaf extract seemed to be more effective in deteriorating oxidative stress and antioxidant defense system in both heart and testis, the latex produced more deleterious effects on the testicular function and sex hormones’ levels. So, the plant latex and ethanolic extract are considered toxic and they may be suggested as rodenticides at 1/10 and 1/20 of LD_50_ which are more or less similar to the reference pesticide, ABM.
